# Aging-Related Vascular Inflammation: Giant Cell Arteritis and Neurological Disorders

**DOI:** 10.3389/fnagi.2022.843305

**Published:** 2022-04-12

**Authors:** Ryu Watanabe, Motomu Hashimoto

**Affiliations:** Department of Clinical Immunology, Osaka Metropolitan University Graduate School of Medicine, Osaka, Japan

**Keywords:** giant cell arteritis, inflammation, neurological aging, vascular aging, vasculitis

## Abstract

Aging is characterized by the functional decline of the immune system and constitutes the primary risk factor for infectious diseases, cardiovascular disorders, cancer, and neurodegenerative disorders. Blood vessels are immune-privileged sites and consist of endothelial cells, vascular smooth muscle cells, macrophages, dendritic cells, fibroblasts, and pericytes, among others. Aging also termed senescence inevitably affects blood vessels, making them vulnerable to inflammation. Atherosclerosis causes low-grade inflammation from the endothelial side; whereas giant cell arteritis (GCA) causes intense inflammation from the adventitial side. GCA is the most common autoimmune vasculitis in the elderly characterized by the formation of granulomas composed of T cells and macrophages in medium- and large-sized vessels. Recent studies explored the pathophysiology of GCA at unprecedented resolutions, and shed new light on cellular signaling pathways and metabolic fitness in wall-destructive T cells and macrophages. Moreover, recent reports have revealed that not only can cerebrovascular disorders, such as stroke and ischemic optic neuropathy, be initial or coexistent manifestations of GCA, but the same is true for dementia and neurodegenerative disorders. In this review, we first outline how aging affects vascular homeostasis. Subsequently, we review the updated pathophysiology of GCA and explain the similarities and differences between vascular aging and GCA. Then, we introduce the possible link between T cell aging, neurological aging, and GCA. Finally, we discuss therapeutic strategies targeting both senescence and vascular inflammation.

## Introduction

Aging is a multifactorial phenomenon that affects virtually every organ system in the human body and is characterized by the progressive functional decline of those organs (Borgoni et al., 2021). Not only infectious diseases like COVID-19, shingles, and pneumonia, but also malignant neoplasms and vascular diseases increase in frequency with aging. Atherosclerosis is a prototypical form of vascular aging (Ungvari et al., 2018; Tyrrell and Goldstein, 2021), and recent studies have revealed the involvement of immune cells and low-grade inflammation in atherosclerosis, known as “inflammaging” (Ferrucci and Fabbri, 2018; Franceschi et al., 2018).

On the other hand, many autoimmune diseases are common in young to middle-aged women, with an exception that is exclusively found in the elderly, giant cell arteritis (GCA) (Weyand and Goronzy, 2014). GCA is classified as large vessel vasculitis (LVV) and affects the aorta and its major branches (Pugh et al., 2022). Large vessel involvement can be complicated by aortic dissection and aneurysm formation, while inflammation of medium-sized arteries causes headache, jaw claudication, loss of vision, and stroke. Moreover, extravascular manifestations —such as fever, malaise, weight loss, polymyalgia rheumatica—also frequently occur (Buttgereit et al., 2016; Yamaguchi et al., 2022). The currently available treatments for GCA include glucocorticoids and tocilizumab (TCZ), an IL-6 receptor inhibitor. TCZ reduces flare-up of GCA and has steroid-sparing effect (Stone et al., 2017); however, discontinuation of TCZ almost inevitably leads to flare-up of GCA, suggesting that TCZ alleviates symptoms, but it does not cure the disease (Quinn et al., 2021). Therefore, further elucidation of the immunopathogenesis of GCA is essential.

This mini-review summarizes the pathological mechanism of vascular aging. Then, the updated immunopathogenesis of GCA is presented and the similarities and differences between vascular aging and GCA are discussed. Then, we introduce the link between T cell aging, neurological aging, and GCA. Finally, possible therapeutic strategies targeting senescence and vascular inflammation are discussed.

## Vascular Aging and Giant Cell Arteritis

### Vascular Aging

**FIGURE 1:**
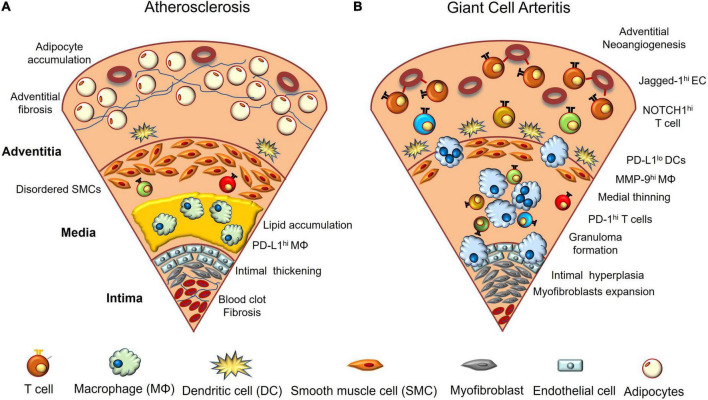
Vascular aging and giant cell arteritis. **(A)** In aged vasculature, lipids accumulate between the intima and media, and macrophages and foam cells phagocytose them. Vascular endothelial cells are multilayered, and blood clots composed of red blood cells and platelets adhere to the luminal surface of the intima. Fibrosis and calcification are often observed. The arrangement of vascular smooth muscle cells becomes irregular and adipocytes accumulate in the adventitial layer. **(B)** GCA-affected arteries are characterized by infiltration of macrophages and activated CD4^+^ T cells. NOTCH1 ligand (Jagged-1)-expressing endothelial cells interact with NOTCH1 expressed on CD4^+^ T cells in the adventitia and polarize T cell differentiation toward Th1 and TH17. Macrophages and multinucleated giant cells release excess proteolytic enzymes such as matrix metalloprotease-9 (MMP-9), disrupting the external elastic membrane, and infiltrated T cells also release various cytokines. As a result, the media becomes thin and the inner elastic membrane collapses. In the intima, myofibroblasts overgrow and block the lumen of blood vessels. Angiogenesis is prominent in the adventitia. Dendritic cells present at the border of the adventitia and media have decreased PD-L1 expression; however, activated T cells highly express PD-1.

Vascular aging refers to the cellular and functional changes that occur in the vasculature during aging, and accounts for most of the morbidity and mortality in the elderly (Figure 1A). Aging-induced functional and structural alterations of the vasculature contribute to not only cardiovascular disease, but also a wide range of age-related diseases, such as cognitive impairment, Alzheimer’s disease, sarcopenia, and kidney dysfunction (Ungvari et al., 2018). A variety of pathophysiological mechanisms drive vascular aging: reactive oxygen species (ROS), mitochondrial dysfunction, inflammation, cellular senescence, genomic instability, increased apoptosis, epigenetic alterations, and clonal hematopoiesis of indeterminate potential (CHIP) (Jaiswal et al., 2017; Ungvari et al., 2018). Among these, mitochondrial dysfunction may play a pivotal role in the vascular aging process (Ungvari et al., 2018; Tyrrell et al., 2020). Impaired mitochondrial biogenesis associated with excess ROS production promotes cellular senescence in endothelial cells (ECs) and vascular smooth muscle cells (VSMCs). Senescent ECs and VSMCs have an increased pro-inflammatory secretome called senescence-associated secretory phenotype (SASP), and this further enhances pathological remodeling of the extracellular matrix and disrupts barrier function in ECs.

CHIP is a relatively new concept in atherosclerosis. With aging, the risk of somatic mutations increases in hematopoietic stem cells residing in bone marrow (Jaiswal et al., 2017). An expansion of hematopoietic clones carrying somatic mutations —most frequently loss-of-function alleles in the genes *DNMT3A*, *TET2*, and *ASXL1*—in the absence of any other hematologic abnormalities is defined as CHIP (Jaiswal et al., 2017). During aging, monocytes that carry such mutations are recruited from the lumen of the blood vessel to the atherosclerotic plaque and there produce excess IL-6, IL-1β, and chemokines (Tyrrell and Goldstein, 2021).

Metabolic reprogramming has also been shown to be involved in vascular aging (Shirai et al., 2016). Macrophages from patients with coronary artery disease (CAD) have an enhanced glycolytic flux as well as increased activity of the tricarboxylic acid cycle, leading to an overproduction of mitochondria-derived ROS. This, in turn, promotes dimerization of the glycolytic enzyme pyruvate kinase M2 and enables its nuclear translocation, boosting IL-6 production (Shirai et al., 2016). At the same time, such metabolically reprogrammed macrophages from CAD patients show increased expression of PD-L1, an immunoinhibitory checkpoint molecule, making CAD patients vulnerable to herpes zoster (Watanabe et al., 2017b, 2018a). PD-L1 expression is regulated by pyruvate, an intermediate metabolite of glycolysis. Immunostaining performed in humans demonstrated that PD-L1 is highly expressed in macrophages infiltrating the vessel wall from the early stage of atherosclerosis (Watanabe et al., 2017b). Thus, metabolic reprogramming in CAD macrophages exacerbates vascular inflammation via IL-6 production, while exerting an immunosuppressive function through PD-L1 expression.

A recent single-cell analysis of human carotid atherosclerotic plaque demonstrated multiple cellular activation—such as ECs, VSMCs, T cells, B cells, and myeloid cells— and mutual activation between cell types (Depuydt et al., 2020). This study not only identified EC subsets with angiogenic capacity and endothelial to mesenchymal transition, but also revealed 2 populations of macrophages; pro-inflammatory macrophages with excess production of IL-1β and tumor necrosis factor and fibrosis-promoting macrophages. Thus, the complex interaction of a wide variety of immune cells and vascular cells shapes the pathogenesis of vascular aging.

### Giant Cell Arteritis

In recent years, great progress has also been made in the understanding of the pathophysiology of GCA. Research has identified the pivotal roles of vascular dendritic cells (DCs) and microvascular ECs in the adventitia in initiating and exacerbating vascular inflammation (Watanabe et al., 2017c; Wen et al., 2017; Zhang et al., 2017). Studies have also elucidated cellular signaling pathways and metabolic fitness in vasculitogenic T cells and macrophages (Watanabe et al., 2018a,b; Zhang et al., 2018, 2019).

The vasculitogenic response is initiated by vascular DCs which reside in the media-adventitial border (Figure 1B). When vascular DCs receive an external danger signal via Toll-like receptor, they upregulate costimulatory molecules, such as CD80 and CD86, and release chemokines. This, in turn, recruits monocytes and CD4^+^ T cells mainly from vasa vasorum located in the adventitia (Ma-Krupa et al., 2004). The monocytes differentiate into tissue macrophages, phagocyte cell debris, and release cytokines, chemokines, and growth factors—such as vascular endothelial growth factor (VEGF)—to further enhance inflammation and neoangiogenesis (Kaiser et al., 1999). At the same time, they form multinucleated giant cells and digest extracellular matrix by releasing proteolytic enzymes, such as matrix metalloproteinase (MMP)-2 and MMP-9, disrupting the elastic membranes (Rodriguez-Pla et al., 2005; Watanabe et al., 2018b; Weyand et al., 2019). Such tissue-destructive macrophages enable activated T cells to infiltrate into otherwise immune-privileged sites and form granulomas. Activated CD4^+^ T cells are dependent on an increased activity of Janus kinase-signal transducer and activator of transcription (JAK-STAT) pathway (Zhang et al., 2018; Vieira et al., 2022) and release multiple cytokines, including IFN-γ, IL-17, IL-21, IL-9, and IL-22 (Deng et al., 2010; Ciccia et al., 2015; Watanabe et al., 2017a; Zerbini et al., 2018). Such a cytokine milieu transforms ECs, VSMCs, and fibroblasts into myofibroblasts, which leads to the occlusion of the blood vessels (Weyand and Goronzy, 2013; Parreau et al., 2021).

The innermost ECs of vasa vasorum control the entry of immune cells. VEGF, which is primarily derived from macrophages and enriched in GCA plasma (Baldini et al., 2012), not only promotes neoangiogenesis in the adventitial layer, but also upregulates the expression of the NOTCH1 ligand on microvascular ECs (Wen et al., 2017). The NOTCH1 ligand stimulates NOTCH1 receptor, expressed on GCA CD4^+^ T cells, and this shifts T cell differentiation toward Th1 and Th17 via activation of mammalian target of rapamycin (mTOR). Thus, microvascular ECs play an unexpected role in instructing T cell differentiation in GCA. Therefore, inhibition of NOTCH signaling or mTOR activation could be a new therapeutic strategy for this disease. High mTOR activity in GCA CD4^+^ T cells is, in part, regulated by CD28 signaling, a “second signal” from antigen-presenting cells (Zhang et al., 2019). For this reason, blocking CD28 signaling could serve as an alternative option to suppress vasculitis.

The next noteworthy research revealed the lack of a system that suppresses aberrant immune activation in vascular lesions. Particularly, deficient expression of PD-L1 is a hallmark feature of vascular DCs present in temporal arteries (Watanabe et al., 2017c; Zhang et al., 2017). Surprisingly, vascular DCs residing in the temporal arteries and monocyte-derived DCs share the feature. PD-L1-deficient DCs have increased capacity for activating CD4^+^ T cells as well as skew naïve CD4^+^ T cell differentiation toward inflammatory phenotypes, such as Th1, Th17, and IL-21-producing T cells. Inhibition of the PD-1/PD-L1 interaction in a preclinical vasculitis mouse model exacerbated the pathology of GCA and recapitulated the intimal hyperplasia and neoangiogenesis in the adventitia, implicating this inhibitory mechanism as an essential regulator of vascular remodeling (Watanabe et al., 2017c; Zhang et al., 2017). Another group confirmed the decreased PD-L1 expression on vascular DCs during vascular inflammation using a mouse model (Sun et al., 2022). Further elucidation of the regulatory mechanism of PD-L1 expression on DCs is necessary.

Recently, attention has been focused on the role of neutrophils in GCA. Neutrophils phagocytose and subsequently kill microorganisms efficiently (Burn et al., 2021). In addition, they unleash their cellular contents into the extracellular space, even post-mortem. In GCA, apoptosis-resistant immature neutrophils produce high levels of ROS, disrupting the endothelial barrier and increasing vascular permeability (Wang et al., 2020).

### Similarities and Differences Between Vascular Aging and Giant Cell Arteritis

Although the disease mechanisms of GCA and CAD may differ, there are significant overlaps between the two. In both diseases, increasing age is the strongest risk factor, and CD4^+^ T cells and macrophages produce excessive cytokines and chemokines in the vascular lesion, resulting in neoangiogenesis in the adventitia and intimal hyperplasia (Figure 1). However, GCA is more tissue-destructive, with macrophages and multinucleated giant cells destroying vascular structures. As a result, the VSMCs undergo cell death and the medial layer becomes thinner. In contrast, in atherosclerosis, the VSMCs proliferate and become irregularly arranged. Arterial stiffness increases due to the accompanied calcification and fibrosis, and adipocyte accumulation is more prominent in atherosclerosis (Tyrrell and Goldstein, 2021).

The direct link between these two diseases is missing. Rather, epidemiological studies have demonstrated that the incidence of GCA was inversely correlated with cardiovascular risk factors, such as obesity, smoking, hyperglycemia, and hypercholesterolemia (Tomasson et al., 2019; Wadstrom et al., 2020). This is consistent with our clinical experience that GCA-positive temporal artery biopsies show little atherosclerosis; while GCA-negative temporal artery biopsies often show atherosclerosis. The exact mechanism behind how vascular aging protects against GCA development remains unclear. One possibility is that, since high blood glucose upregulates PD-L1 expression on macrophages (Watanabe et al., 2018a), both tissue macrophages in atherosclerotic plaque and monocyte-derived macrophages from CAD patients overexpress PD-L1 and fail to support clonal expansion of CD4^+^ T cells (Watanabe et al., 2017b). Impaired T cell immunity due to hyperglycemia may suppress the excess inflammatory response, as seen in GCA.

### T Cell Aging and Giant Cell Arteritis

**FIGURE 2:**
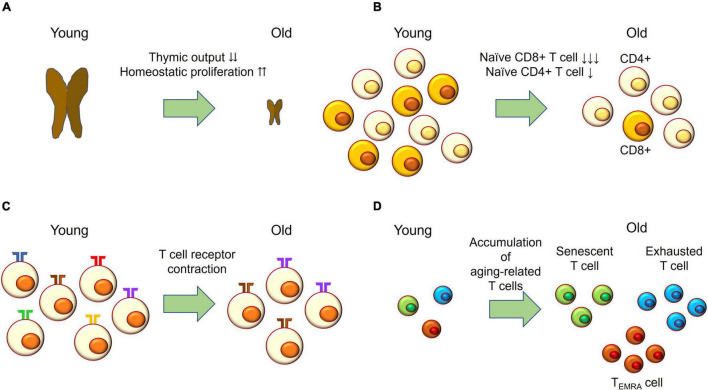
T cell aging. **(A)** With age, thymic output drastically decreases and the naïve T cell compartment is maintained by homeostatic proliferation. **(B)** Naïve CD4^+^ T cells slightly decrease in absolute number with age; while naïve CD8^+^ T cells drastically decrease with age. **(C)** The number of T cell receptors—also called the T cell repertoire—declines with age, while clonal sizes increase with age. This process is called T cell receptor contraction. **(D)** T effector memory CD45 RA (TEMRA) cells; exhausted T cells expressing PD-1, TIM-3, and LAG-3; and senescent T cells accumulating with age.

In contrast to vascular aging, T cell aging may participate in the pathogenesis of GCA (Figure 2). With aging, thymic involution accelerates homeostatic proliferation of naïve T cells (Figure 2A); however, naïve T cells, particularly naïve CD8^+^ T cells, fail to maintain their absolute number (Figure 2B). Variation of T cell receptors is also reduced, while clonal sizes increase (Figure 2C). In addition, aging-related T cells—such as senescent T cells, exhausted T cells, and T effector memory CD45 RA (TEMRA) cells—accumulate (Figure 2D). Senescent T cells show irreversible cell cycle arrest but have SASP and release pro-inflammatory cytokines. Exhausted T cells express programmed death-1 (PD-1), TIM3, and LAG-3, and their effector functions are defective. TEMRA cells have short telomeres and show cell cycle arrest while maintaining high effector function (Goronzy and Weyand, 2017).

Although the direct evidence showing T cell aging in GCA is lacking, some indirect evidence does exist. Firstly, the T cell repertoire in GCA vascular lesions is restricted, while clonal sizes are expanded (Weyand et al., 1994). Secondly, CD4^+^ T cells have an increased capacity for cytokine production. Thirdly, granulomatous inflammation in Takayasu arteritis—another type of LVV seen in young women—is composed of macrophages, CD4^+^ T cells, and CD8^+^ T cells; while CD8^+^ T cells are rarely seen in GCA (Watanabe et al., 2020a,b). Further studies are needed to determine whether T cell aging accelerates vascular pathology in GCA.

## Neurological Aging and Giant Cell Arteritis

### Neurological Aging

Dementia and neurodegenerative disorders are now a major problem in public health. In 2018, nearly 50 million people were affected by Alzheimer’s disease and other dementias all over the world (Grande et al., 2020). Dementia and neurodegenerative disorders are prototypes of neurological aging, with aging being the strongest risk factor for these diseases (Hou et al., 2019). Pathways implicated in the development and progression of these disorders include brain resilience, vascular damage, neuroinflammation, oxidative stress, dysfunctional autophagy, apolipoprotein homeostasis, and cellular senescence (Baker and Petersen, 2018; Grande et al., 2020; Montagne et al., 2020). Evidence is accumulating that various cell types in the neural network, including neurons, oligodendrocytes, astrocytes, microglial cells, and endothelial cells show senescent phenotypes with age. Commonly observed features of senescent cells include cell cycle arrest, telomere shortening, resistance to apoptosis, and the SASP, just like in peripheral tissues (Baker and Petersen, 2018).

### Neurological Aging and Giant Cell Arteritis

Well-known neurological complications of GCA include stroke, cerebral infarction, and ischemic optic neuropathy. Cerebrovascular accident (CVA) is not specific to GCA, and does not have high diagnostic accuracy in GCA (van der Geest et al., 2020). However, patients with GCA are more likely to develop CVAs than age- and sex-matched controls (Robson et al., 2016; Tomasson et al., 2019), particularly within 1 year from diagnosis. This may be explained by strong vascular inflammation and high-dose glucocorticoids. The risk factors for CVA include increasing age, male, and social deprivation in GCA (Robson et al., 2016).

In addition, the evidence is accumulating that other forms of neurological disorders, such as dementia and neurodegenerative disorders, can be initial or coexistent manifestations of GCA (Pascuzzi et al., 1989; Caselli, 1990; Caselli and Hunder, 1993; Ely, 1998; Incalzi et al., 2005; Alisky, 2008; Solans-Laqué et al., 2008; Kushida et al., 2011; Lahaye et al., 2020). It is noteworthy that some of these cases showed improvement after GCA treatment, indicating that GCA could manifest as a “treatable” neurological disorder. Blood sampling and imaging may be useful in diagnosing GCA in patients with dementia, who have difficulty in reporting symptoms (Kushida et al., 2011).

More recently, an epidemiological study has demonstrated that neurodegenerative disorders accounted for 11% of deaths in GCA patients (Chazal et al., 2018). This suggests that the prevalence of neurodegenerative disorders in patients with GCA could be much higher. Such manifestations had been underestimated or considered rare, but this may be no longer the case for patients with GCA. Glucocorticoids and other treatments may not be sufficient to control neurological aging due to vascular damage from GCA or that patients with GCA are able to live longer than before and develop neurodegenerative diseases during the disease process.

## Discussion

We have focused on the relationship between aging in various organ systems and GCA. Is there a way to suppress both senescence and vascular inflammation? As described earlier, since vascular aging is characterized by low-grade inflammation, several attempts have been made to prevent the recurrence of cardiovascular events by controlling inflammation. A large clinical study showed that controlling inflammation by blocking IL-1β could suppress atherosclerosis and reduce cardiovascular events (Ridker et al., 2017); however, subsequent research revealed that IL-1β has atheroprotective effects in mice (Gomez et al., 2018), which suspended further clinical research.

Are there any ways to inhibit cellular senescence? Three strategies for targeting cellular senescence have been proposed (Ovadya and Krizhanovsky, 2018). The leading option is to induce apoptosis selectively in senescent cells with senolytic drugs. The second approach is to potentiate an immune response against senescent cells, leading to apoptotic cell death. The third one is selective blockade of SASP (Ovadya and Krizhanovsky, 2018). Among those therapies, inhibitors of JAK-STAT pathway are not only expected to be effective against cellular senescence by suppressing excessive cytokine signaling, but also expected for treating GCA (Zhang et al., 2018; Rathore et al., 2022; Vieira et al., 2022). In addition, recent research has shown that the complement C3a receptor, expressed on ECs, promotes dysfunction of blood brain barrier as well as vascular inflammation during aging, leading to neurodegenerative disease (Propson et al., 2021). Targeting complement activation could also be a novel therapeutic approach for the treatment of brain aging, neurodegenerative disorder, and vascular inflammation.

In conclusion, GCA is not just vascular aging, but vascular inflammation with the involvement of T-cell and neurological aging. Drugs that could simultaneously inhibit cellular senescence and vascular inflammation may be useful.

## Author Contributions

RW drafted the manuscript. MH revised and finalized the manuscript. Both authors contributed to the article and approved the submitted version.

## Conflict of Interest

The authors declare that the research was conducted in the absence of any commercial or financial relationships that could be construed as a potential conflict of interest.

## Publisher’s Note

All claims expressed in this article are solely those of the authors and do not necessarily represent those of their affiliated organizations, or those of the publisher, the editors and the reviewers. Any product that may be evaluated in this article, or claim that may be made by its manufacturer, is not guaranteed or endorsed by the publisher.
